# Measuring electro-adhesion pressure before and after contact

**DOI:** 10.1038/s41598-023-38872-6

**Published:** 2023-07-20

**Authors:** Sylvain Schaller, Herbert Shea

**Affiliations:** Soft Transducers Laboratory, Ecole Polytechnique Fédérale de Lausanne (EPFL), Rue de la Maladière 71b, 2000 Neuchâtel, Switzerland

**Keywords:** Engineering, Materials science

## Abstract

Electro-adhesion (EA) is a low-power, tunable, fast and reversible electrically-controlled adhesion method, effective on both conducting and insulating objects. Typically, only the electro-adhesive detachment force, i.e., the force required to separate an object from the EA patch, is measured. Here, we report a method that enables comparing the pre-contact EA attachment forces with post-contact EA detachment forces. We observe that pre-contact pressures are 1 to 100 times lower than post-contact detachment pressures, indicating the large role played by surface forces, charge injection, and polarization inertia. We characterize the time-dependence of pre- and post-contact EA forces as a function of the applied voltage waveform, observing that using an AC drive allowing for much faster release than DC operation. We measure both EA forces on conductive and insulating objects, using over 100 different EA patches covering a wide range of electrode dimensions. At 400 V, the EA release pressures for conductive objects range from 1 to 100 kPa, and are 1 to 10 times higher than pre-contact adhesion force. For dielectric objects, release pressures are 1 to 100 higher than pre-contact adhesion pressures. The methodology presented in this paper can enable standardized EA characterization while varying numerous parameters.

## Introduction

**Figure 1:**
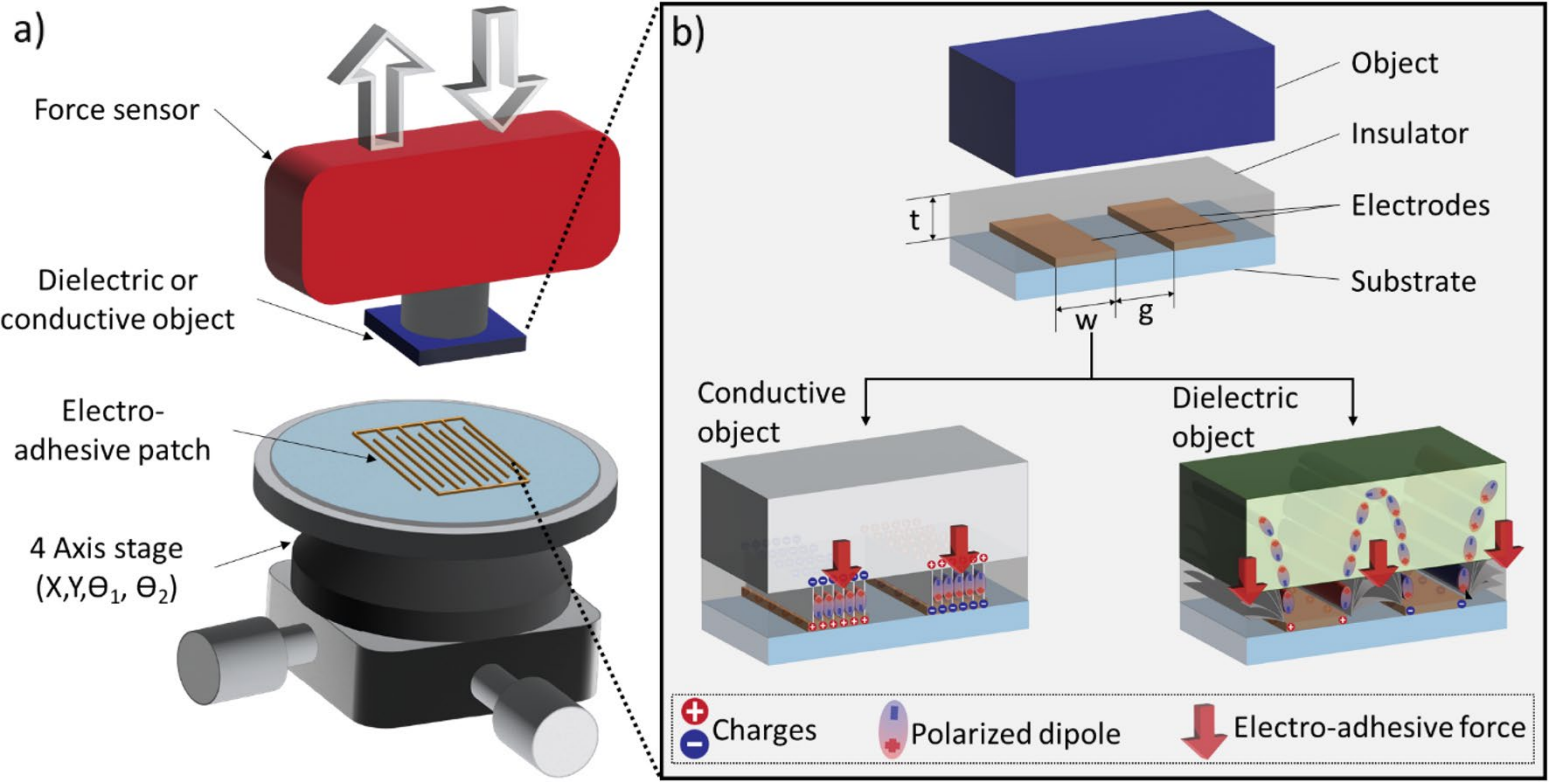
(**a**) EA test bench consisting of a force sensor, a target object (conductive or dielectric), an EA patch with interdigitated electrodes, and a multi-axis stage (**b**) Electroadhesion for conductive and dielectric objects. The EA force (red arrow) is normal to the EA patch in both cases, but the electric field distributions are different, with fringing fields playing an essential role in the case of a dielectric object.

Electro-adhesion (EA) is an electrically-controlled electrostatic attraction between two objects. There are two main EA architectures. In the first, one applies a voltage directly between two conductive objects, for instance between a silicon wafer and a metal plate. This was formalized in the early 20th century by Johnsen and Rahbek (the Johnsen–Rahbek effect)^1^. In a second configuration, one does not apply a voltage to the object one seeks to adhere to, but only to interdigitated electrodes patterned on an EA patch, covered by a thin insulator layer to avoid short-circuiting the electrodes. The electric fields from the patch polarize or charge the object, leading to an EA force between the patch and the object (see Fig. 1). This interdigitated structure has been used for a wide range of grippers and robotic applications, as reviewed recently by Guo et al.^2^ and Rajagopalan et al.^3^.

A typical EA test bench, illustrated in Fig. 1a, includes an EA patch with interdigitated electrodes, a force sensor to which a target object is attached, a vertical linear translation stage, and a tip/tilt platform to ensure parallelism. The setup measures the EA force between the EA patch and the target object, which can be either conductive or a dielectric. Figure 1b schematically illustrates the electrostatic interaction for both cases. For a grounded conductive object, the electro-adhesive force due to an interdigitated EA patch can be described as a simple electrostatic force between parallel plates, ignoring fringing fields. For dielectric objects, the fringing electric fields from the electrodes polarize both the object and the insulator layer. The polarization of the object leads to an electrostatic attraction. The electric field distribution depends on the geometry and the permittivities of the insulator and of the object, and on the time evolution of the polarization.

Literature values for the pressure to detach the EA patch from an object range from 0.1 kPa to 100 kPa for conductive objects^4–10^ and for dielectric objects^4,5,7,9,11–18^. The very large spread in reported EA pressures is in part due to the many relevant parameters for EA. Depending on the application, research groups report either normal forces or shear EA forces. The objects being adhered to can be conductive or insulating, with different surface roughnesses. Different electrical insulating materials can be used to passivate the electrodes, which can have different surface topographies. The applied voltage can be DC or AC. Several groups report hybrid devices, combining dry adhesion (e.g., Gecko-inspired adhesives) with electroadhesion^5,9,12^.

### Standard EA measurements methods

EA has been used extensively for soft grippers, as pioneered by Monkman^19,20^, enabling grasping without squeezing, making it particularly well suited for manipulating delicate objects^9,11,19–23^. Numerous applications of electro-adhesive technology in robotics have been reported, including grasping, crawling and climbing^2,11^.

**Figure 2:**
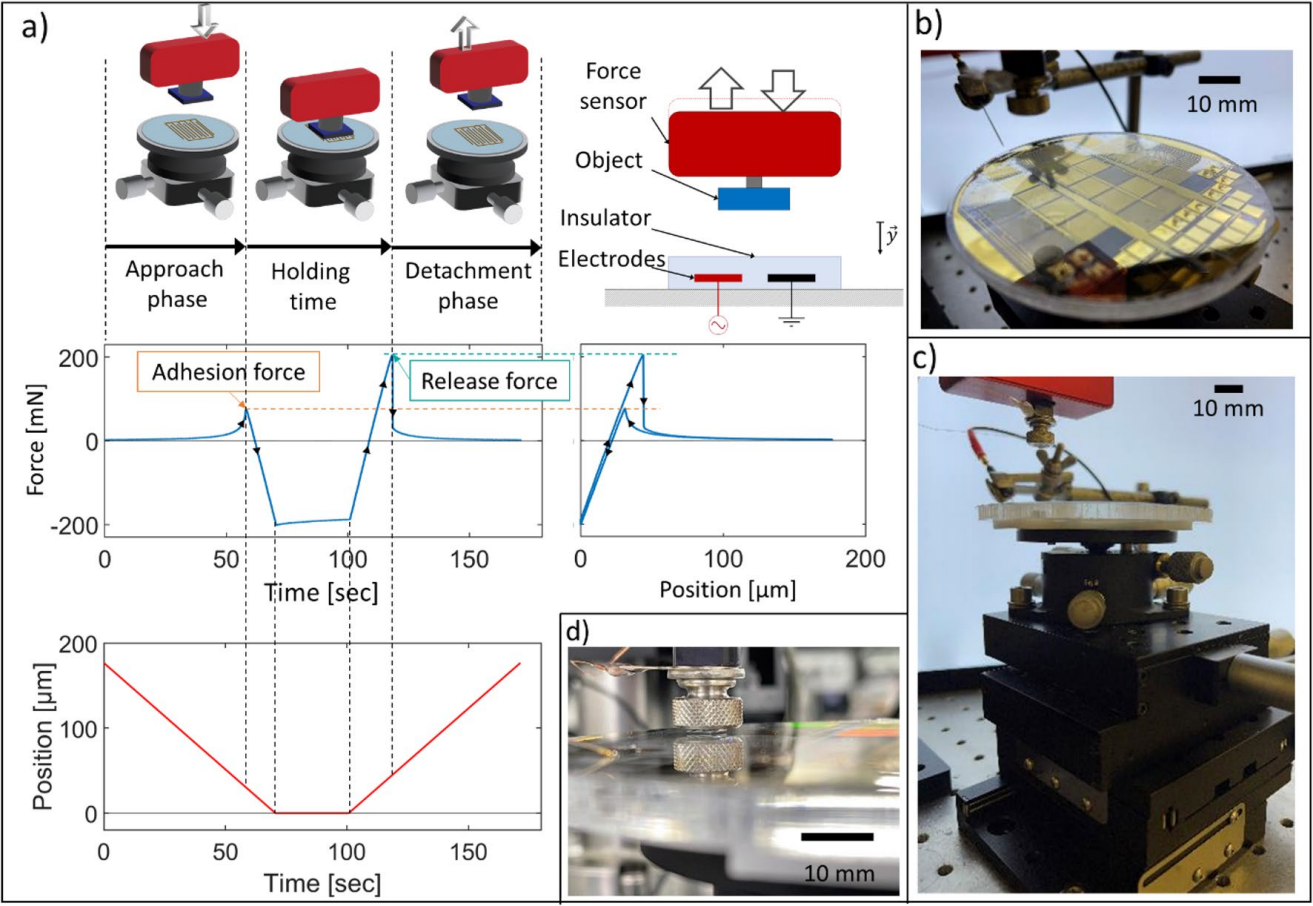
(**a**) Three main phases when measuring EA pressure: the approach, holding, and detachment. A typical force versus time plot, the corresponding force versus position plot and the position of the sensor versus time plot are shown. The positive force peaks correspond to the adhesion and release forces. We refer to a positive force as attractive; a negative force as repulsive. The difference between adhesion and release forces is due to charge injection, contact forces and polarization inertia. (**b**) Photograph of a fabricated wafer containing EA patches with different dimensions, mounted on our test bench (**c**) Side view of our test bench (**d**) Close-up of the sensor approaching an EA patch.

How authors chose to characterize EA performance has often been dependent on their application, e.g., does one aim for shear or for normal forces, is release time an important metric, are the EA patches rigid or flexible, etc. Regardless, methods to measure EA forces share several common steps. As described in Fig. 2a, a typical process for measuring normal forces consists in (a) an approach phase to bring the EA patch into contact with the object, (b) a contact phase, and finally (c) the detachment phase, during which the release force is measured. During the contact phase, a wait time of 10 s, 60 s and 90 s is often used^5,7,9,14^. This holding time has a significant impact on release EA pressure, greatly increasing the holding force, but slowing the release when the voltage is turned off. The holding times correspond to the most obvious use cases for EA patches in grippers^9,17,18,21^ and as adhesive surfaces for robotics^6,15,16,24^. A force relaxation of order 20 mN is observed during the holding time, which we attribute primarily to the viscoelasticity of the insulating layer between the interdigitated electrodes and the object^25^. Figure 2b, c, and d depict various photographs of the fabricated wafer, the test bench, and the object on the force sensor.

The “electro-adhesive pressure” reported in all papers to date is the release pressure. We extend here the measurement process to record force data also before contact between the EA patch and the object, during the holding time phase, in addition to the commonly reported detachment phase. We can thus measure both (i) the pre-contact adhesion force, which is the maximum force while the object approaches the EA patch, and (ii) the release force, the maximum force when detaching the object from the EA patch. We observe EA release pressures between 1 to 10 times higher than adhesion pressures for conductive objects, and 1 to 100 times higher for dielectric at 400 V. Surface contact forces, charge injection, and slow polarization can be much larger than the “pure” EA force, which is the only force we measure in the pre-contact case.

## Methods

We carry out two sets of measurements. The first study, “Effect of Waveform and Holding time”, investigates the impact on both the adhesion and the release pressure of AC and DC waveforms with holding times of 0, 10, 30 and 90 s, for one given dimension of electrodes. The second study, “Adhesion versus Release Pressure”, reports the adhesion pressure and the release pressure, for electrodes driven by a 400 V AC bipolar square wave, for a broad range of electrode dimensions, from 5 µm to 500 µm, with no additional holding time during contact. These measurements were made using the same experimental setup.

### Test bench

The test bench measures the force between fixed EA patch fabricated on a glass wafer and an object (insulating or conductive) moving on a motorized stage. We move the object down until contact (attachment), then pull it up (release). Each measurement cycle thus provides: (a) during the approach: the electro-adhesive force before contact, i.e., avoiding any dependence on tackiness of the insulator and other surface forces, and (b) on the way up: the EA force required to pull the object off of the electrodes.

We measured the EA pressure on more than 180 microfabricated EA patches. To date, the lowest gap and width for EA electrodes was 80 µm, reported by Wang et al.^26^ in 2012. In order to characterize electrodes with smaller dimensions, in view of lowering the operating voltages, and to compare with literature, our interdigitated electrodes have widths and gaps ranging from 5 µm to 500 µm. Our smallest devices are up to an order of magnitude smaller than typical EA structures (see SI section S1).

The EA electrodes are 80 nm thick gold, patterned on 100 mm diameter Borofloat® wafer. Each wafer holds 30 sets of interdigitated electrodes, as shown in Fig. 2c. Microfabrication consisted of metal deposition, photoresist coating, direct laser writing, Argon ion etching, and finally photoresist (PR) stripping (details are provided in SI section S2). After electrode fabrication, we blade casted the insulator P(VDF-TrFE-CTFE) on the wafer, with final insulator thicknesses t of 6 µm and 20 µm. We do not dice the wafers. We chose P(VDF-TrFE-CTFE) (poly vinylidene fluoride, trifluoroethylene, 1,1-chlorotrifluoroethylene) as our insulator due to its high relative permittivity of approximately 40, and because of the high EA forces it enabled in electrostatic clutches^27,28^. This polymer has a breakdown field above 120 V.µm^−1^, which allows us to operate reliably at 400 V even for a thickness of 6 µm.

The objects to be adhere to (both conductive and insulating) used in this study were flat, with sub-µm roughness (see SI section S3). The conductive object was a gold-coated Silicon chip of dimensions 5 mm × 5 mm. The dielectric object was a 5 mm × 5 mm glass chip, diced from a Borofloat® wafer.

The test bench consists of a 4.4 N force sensor (Low Profile Load Cell, LRF400 from FUTEK) attached to a motorized platform enabling vertical translation of the object relative to the wafer. The wafer is clamped on a tip/tilt platform to ensure parallelism with the object, with 0.03° angular resolution. This tip/tilt precision is required because an angular misalignment between the object and the patch leads to significantly lower measured pressure (see SI section S4). An XY stage allows translating the wafer under the force sensors while maintaining angular alignment. The automated motorized z-stage motion ensured repeatability of the measurements.

Electrical connections to the EA patches on the wafer were made using probe needles (see Fig. 2b and c). Current and voltage are measured continuously using an oscilloscope equipped with a high voltage probe. The voltage is supplied by a high voltage amplifier (Trek 609E-6) driven by a signal generator. When comparing AC and DC waveforms, the maximum voltage was 2 kV. The AC signal is a symmetrical bipolar square wave, to minimize dielectric charging while keeping a constant Maxwell pressure in time. For the “Adhesion versus Release Pressure” study, the maximum voltage we used was 400 V (due to breakdown in the smallest gaps at higher voltages).

### Measurement process for pre- and post- contact EA pressure

**Figure 3:**
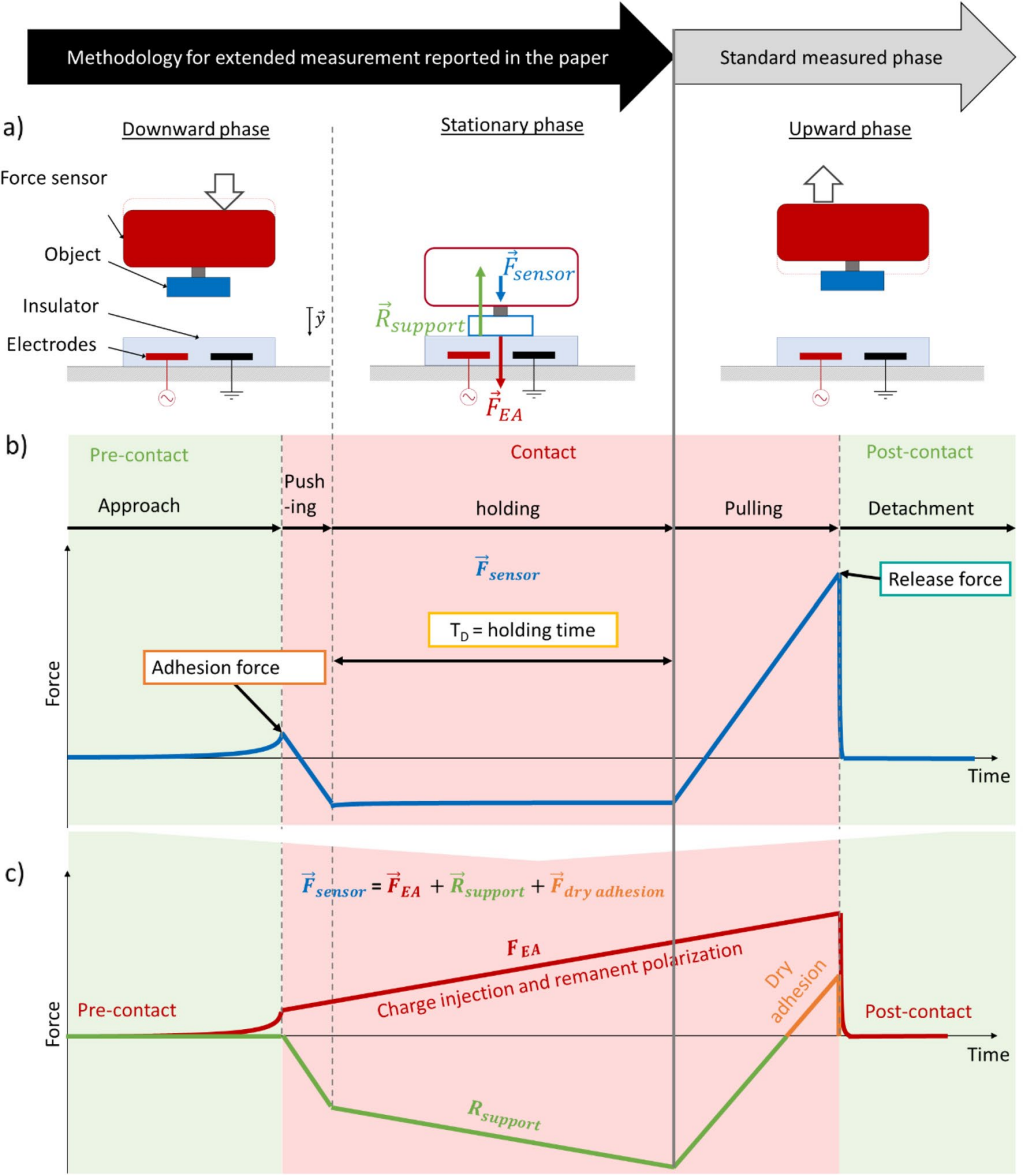
(**a**) Measurement cycle. Center: the forces acting on the object during the holding phase. (**b**) plot of the force measured by the force sensor during the different phases of the measurement cycle. (**c**) Schematic split of the measured force into a “pure” EA pressure, plotted in red, and reaction force and dry adhesion pressure in green and in orange. The adhesion force is purely an EA force, while the release force is the sum of the EA force and dry adhesion. The increase over time of the EA force and support reaction illustrate the increasing effect of charge injection and of remanent polarization.

Our method is designed to measure both adhesion and release force. The EA pressure was computed by dividing the recorded force value by the surface area of the object, which was taken as 25 mm^2^ for both conductive and dielectric objects. As illustrated in Fig. 3a, the object is initially positioned several mm above the EA patch. The measurement cycle consists of three phases. (i) the downward phase: the EA voltage is turned on; the object approaches the EA patch at a constant speed until it contacts the patch. The stage then pushes further down until the force sensor reads 200 mN. (ii) an optional stationary holding phase: the object remains in contact with the patch for a set time (from 0 to 90 s) with the voltage on. (iii) the detachment phase: with the still voltage on, the object is raised up to its original position, with sudden detachment occurring during this phase. Force, voltage and current data are recorded continuously during all phases.

As plotted in Fig. 3b, we see two peaks in EA pressure during the measurement. As the object nears the EA patch during the approach, the electro-adhesive force increases: the object is pulled down towards the EA patch. The positive peak in force is the maximum “contactless” EA force which correspond to the “pure” EA adhesion force. Once the object touches the EA patch, the object (and force sensor) is compressed, leading to a negative force reading. We stop the stage motion when the compressive force reaches  − 200 mN, in order to avoid damaging the patch or object by pushing them too hard together. The exact value of this maximum compressive force (eg 150 mN or 300 mN) has negligible influence on the peak EA forces. We hold the contact position during the holding phase for 0 to 90 s, during which charge injection and slow polarization can occur. The stage is finally raised during the detachment phase. The force decreases in absolute value, passes zero (i.e., becomes attractive, showing adhesion), increases and then suddenly drops to zero once the object detaches from the EA patch. All displacements were at a speed of 3 µm/s.

This measurement method allows quantitatively comparing adhesion and release pressures, and thus distinguishing contact forces from EA forces. We varied several EA parameters including object type, insulating layer thickness, holding time or signal waveform.

### EA pressure data

We define the adhesion pressure as the maximum pressure reached during the downward phase (i.e., pre-contact) and the release pressure as the maximum pressure reached during the upward phase (see Fig. 3b). Figure 3b plots in blue the measured force versus time. In Fig. 3c, we schematically split the measured pressure into a “pure” EA pressure, plotted in red, and reaction force and dry adhesion pressure in green and in orange.

The red plot schematically represents the EA force, which increases during the downward phase as the electric field at the object surface or in the object increases as the object nears the EA patch. Then once in contact, at a constant position, the EA force likely increases due to charge injection, polarization, Johnsen-Rahbek effect and other contact interactions. For simplicity, we assumed a linear increase over time for the figure. Finally, the EA force drops at detachment and becomes null after the object has returned to its initial distant position.

The green plot schematically represents the mechanical reaction force of EA on the object attached to the force sensor. Starting from zero well before contact, this reaction force increases in amplitude (becoming more negative) as the object pushes on the patch. After contact, the slope is proportional to the imposed displacement of the sensor, due to the stiffness of the system (dominated by the stiffness of the force sensor). In the holding stage, the reaction force increases to compensate for the EA force that is increasing due to polarization and charge injection. During the upward phase, the reaction force decreases, then switches sign (becomes attractive). We schematically plot this in orange and label it the dry adhesion force. The reaction force drops to 0 after detachment between the object and the patch.

The adhesion force corresponds to a pressure purely due to electrostatic attraction, i.e., to material polarization in the case of a dielectric object, and to charge mobility in the case of a conductive object. The release pressure includes surfaces forces and electrostatic forces from charge injection.

Data for all tested samples and conditions are available in a Zenodo repository. (https://doi.org/10.5281/zenodo.6417174).

### Experimental conditions for “Effect of Waveform and Holding time”

This study compared the EA pressure for AC and DC waveforms, for both adhesion and release. We measured the electro-adhesive pressure using the same method as described in section "EA pressure data", for voltages from 0 V up to 2 kV.

For the DC case, we added 0, 10, 30 and 90 s holding times T_D_ (i.e., additional contact time). These experiments were performed on a sample with an electrode width of 100 µm, an electrode gap of 100 µm and an insulator thickness t of 20 µm.

Unlike earlier studies of DC residual force characterizations experiments, we report both pre- and post-contact (i.e., approach and detachment) forces, as well as the dependence of the residual forces on the contact duration^29,30^.

### Experimental conditions for “Adhesion versus Release Pressure” measurement at 400 V

The goal of this study is to characterize the EA pressure for two thicknesses of insulating layer for both conductive and dielectric objects, for a broad range of electrode dimensions. We used a maximum voltage of 400 V because for our smallest gaps (5 µm) and thinnest insulator layer (6 µm) the breakdown voltage of the insulator is around 500 V.

To measure the electro-adhesive pressure, each patch was tested at 0 V, then 400 V, and again at 0 V for both conductive and dielectric objects with no holding of contact. For tests on conducting objects, we used a square bipolar drive at 5 Hz. This frequency was chosen to minimize charge injection and thus additional adhesion forces. For dielectric objects, a 1 Hz bipolar waveform was used, to ensure reasonably full/complete polarization of the object. The choice of bipolar frequency is explained in the supporting information section SI5. We performed a total of 511 measurements on conductive objects and 112 for dielectric objects.

## Results

### Effect of Waveform and Holding time

**Figure 4:**
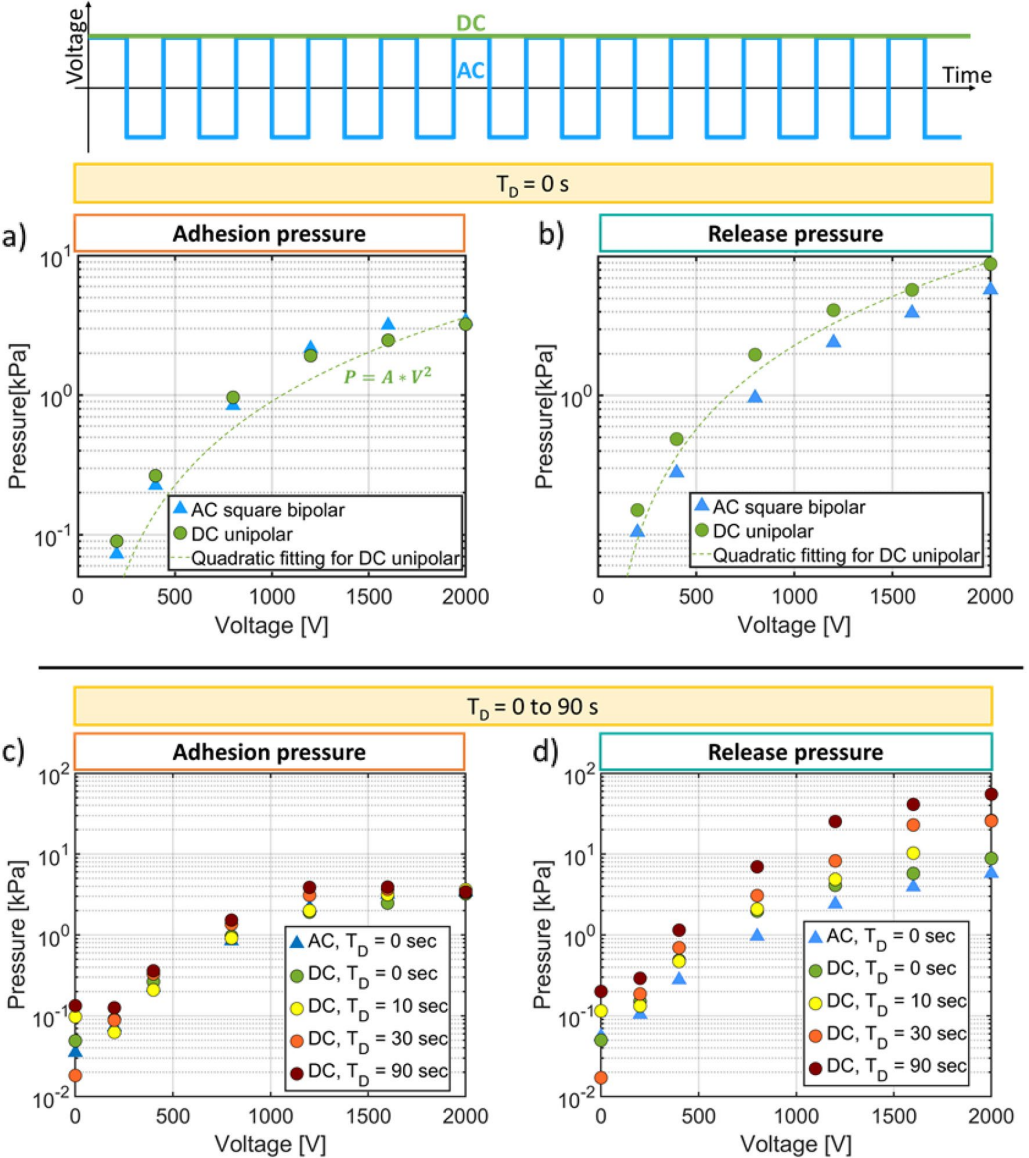
Effect of Waveform and Holding time on the EA pressure measured on a dielectric object using an EA patch with electrode width and gap of 100 µm, and a dielectric thickness of 20 µm. (**a**) and (**b**) Adhesion pressure and Release pressure as a function of voltage and for bipolar AC and DC drive. A quadratic fit of P = A V^2^ to the DC unipolar pressure versus V curve is shown, with A = (0.903 ± 0.17) *10–7 [kPa/V^2^] for adhesion and A = (2.29 ± 0.2) *10–6 [kPa/V^2^] for release. (**c**) and (**d**) Adhesion pressure and Release pressure as a function of voltage and for AC (1 Hz) and for DC drive with holding times of 10, 30 and 90 s.

**Figure 5:**
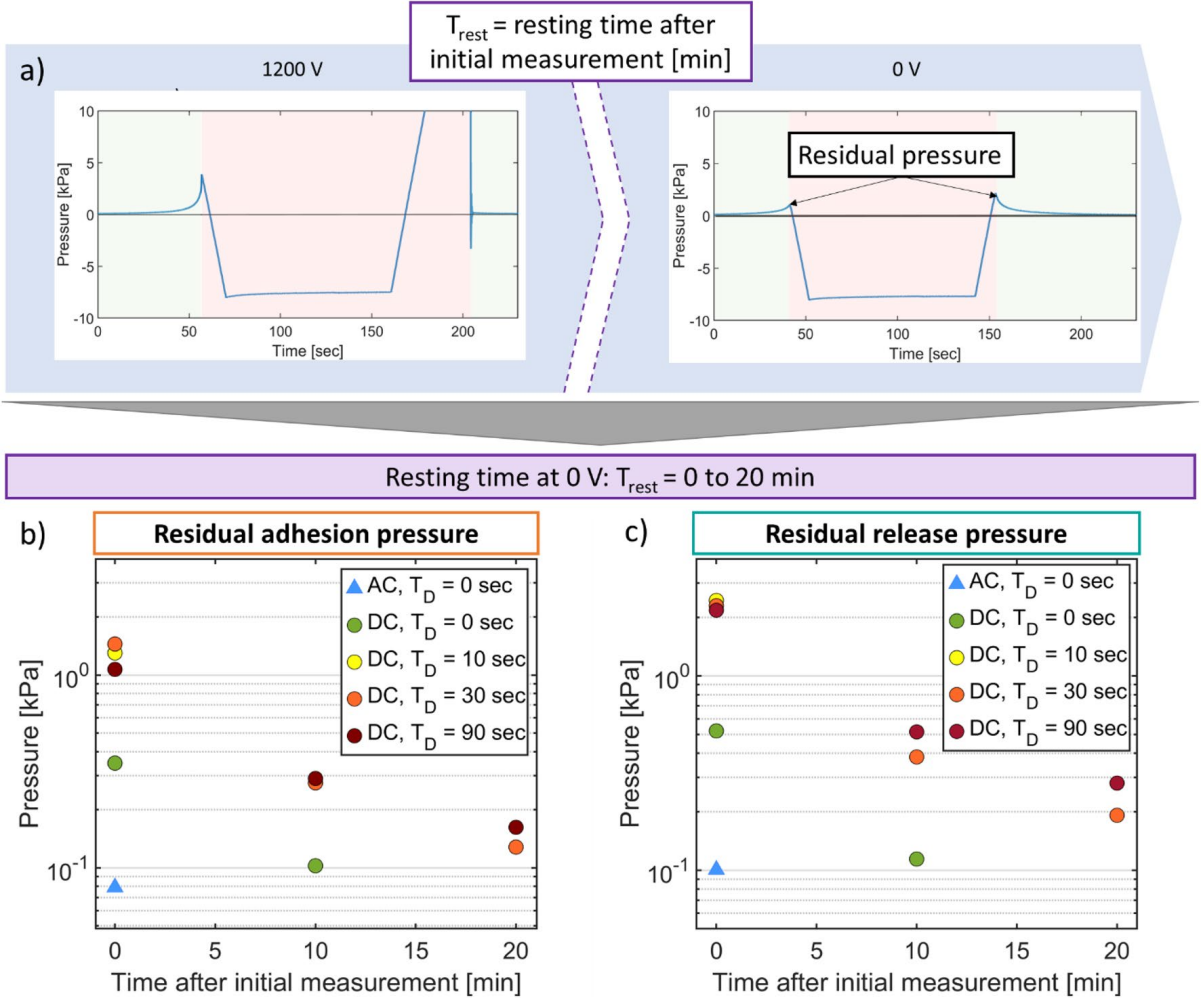
Effect of Waveform and Holding time T_D_: (**a**) Process for measuring the residual EA pressure (i.e., the pressure at 0 V). A force versus time measurement is taken at 1200 V. The same measurement is then repeated immediately at 0 V, to measure the residual pressure. If the residual pressure is higher than 0.2 kPa, the 0 V measurement is repeated 10 min later. (**b**) Residual adhesion pressure taken 0 min, 10 min and 20 min after the measurements at 1200 V. (**c**) Residual release pressure taken 0 min, 10 min and 20 min after the measurements at 1200 V. Data was taken on an EA patch with electrodes width and gap of 100 µm, and a dielectric thickness of 20 µm.

In this section, we compare the EA forces on a dielectric object when driven by a unipolar DC and bipolar square AC waveforms. We also study the effect of adding an addition holding time T_D_ while the object is in contact with the EA patch. The data shown in Figs. 4 and 5 is taken using one single geometry: an interdigitated EA patch with electrodes width of 100 µm, gap of 100 µm and insulator thickness of 20 µm.

Figure 4a and b plot the measured adhesion and release pressures versus voltage for bipolar square AC (1 Hz) and DC waveform with no additional holding time between the approach phase and the detachment phase. For all conditions, the adhesion and release pressures increase with the voltage. A purely capacitive force should theoretically scale as V^2^. The good fit of the measured data to a quadratic curve confirms the V2 dependence of the EA pressure.

No significant difference in adhesion (ie pre-contact) pressures is seen between AC or DC waveforms. This is expected as charge injection cannot occur before contact. Release pressures are 1.5 higher for DC than for AC. The minimum duration of contact between the patch and the object is approximately 24 s, given the time to reach 200 mN of force. During this contact time, charge injection and remanent polarization can occur, more so when using DC. This minimum contact time can explain the higher release pressures observed for DC compared to AC.

Figure 4c and d plot adhesion and release pressures when an additional holding time T_D_ (contact time) of 10, 30 and 90 s is used for the DC cases. The contact time has several effects. Charges can be injected into the insulator due to the high electric field, leading to a higher capacitive pressure. Close contact between the surfaces leads to high Van der Waals forces. Given the polarization inertia, the EA pressure increases over time^31^.

Figure 4c illustrates that the adhesion pressures are very similar for AC waveforms and for the DC waveforms with different hold times, which is expected as no net charge injection occurs before contact. Figure 4d however shows that adding the holding time gives up to a sixfold increase in the release pressure for the DC case. At 2 kV, the release pressure increases from 5 kPa for AC drive up to almost 60 kPa for DC drive when a 90 s holding time is used. The longer the dielectric object remains in contact with the EA patch, the higher the release pressure is.

In Fig. 5, we investigate how long it takes for the increased force stemming from a holding time decay after the voltage is set back to zero. In order to characterize the effect and time dependence of charge injection and object remanent polarization, after each 1200 V measurement, we performed our standard force versus time measurement at 0 V (as illustrated in Fig. 5a). We measured the residual pressure due to charge injection and dielectric object polarization at 0 min, 10 min and 20 min after the measurements at 1200 V, as shown in Fig. 5b and c, for residual adhesion and residual release pressures. At 0 min after the 1.2 kV measurements, residual pressures for AC waveforms are less than 0.1 kPa, while for DC waveforms, residual pressures are five times larger. With a holding time of 10 s or higher, the residual adhesion pressure increases to a maximum of 1.5 kPa and residual release pressure reaches 2.4 kPa. The residual pressure drops to less than 1 kPa after 10 min and become lower than 0.3 kPa after 20 min.

### Adhesion versus release measurements at 400 V for different electrode dimensions

**Figure 6:**
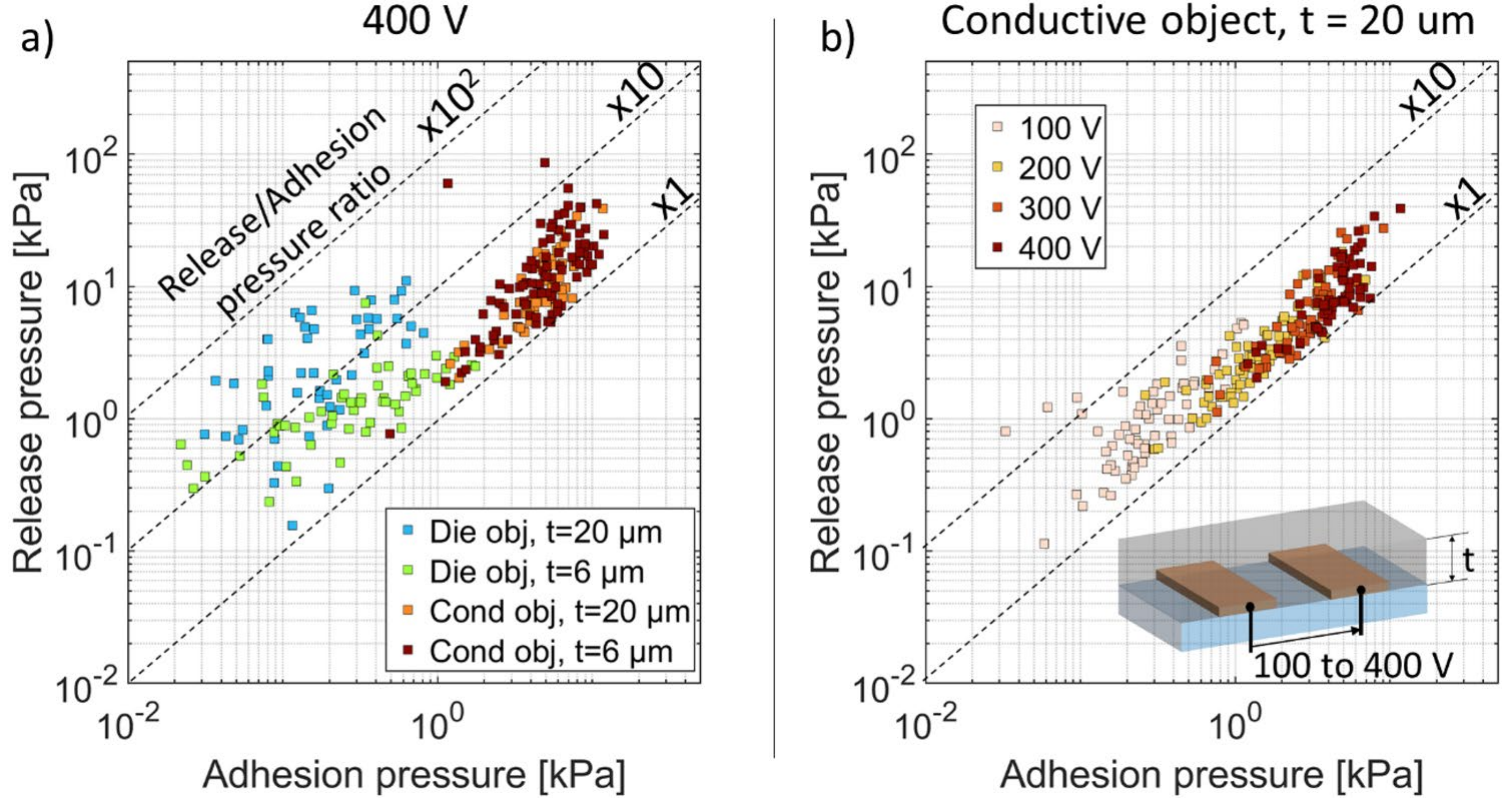
(**a**) Release pressure versus adhesion pressure at 400 V for conductive and dielectric objects, and for EA patches with 6 µm and 20 µm insulator thicknesses. Each data point corresponds to one of the over 180 different EA patches tested. The data clusters for the different cases, despite the large range of electrode dimensions used in this study. Conductive objects have higher EA pressures than dielectric objects. (**b**) Release pressure versus adhesion pressure from 100 to 400 V for a conductive object with an EA patch insulating thickness of t = 20 µm. The data clusters show that increasing the voltage increasing both the adhesion and the release pressure.

Figure 6a plots the adhesion pressure (i.e., pre-contact pressure, with no surface forces) versus the release pressure (i.e., post-contact pressure, with surface forces) at 400 V for over 180 different EA patches, tested on both conductive and dielectric object, and with insulator thickness t of 6 µm and 20 µm. Each of the over 267 data points corresponds to one combination of electrode width and gap (ranging from 5 µm to 500 µm), object type and insulator thickness.

Dashed lines in Fig. 6 show different ratios of release to adhesion pressure. The measured release pressure is always greater than the adhesion pressure (which is expected given the extra forces that are involved after contact is made), and the ratio is lower for conductive objects than for insulating objects.

Figure 6b plots the measured release pressure versus the measured adhesion pressure for applied voltages of 100 V, 200 V, 300 V and 400 V for a conductive object and 20 µm thick insulator. Higher voltages lead to higher forces, as was more clearly shown for a single EA patch in Fig. 5.

**Figure 7:**
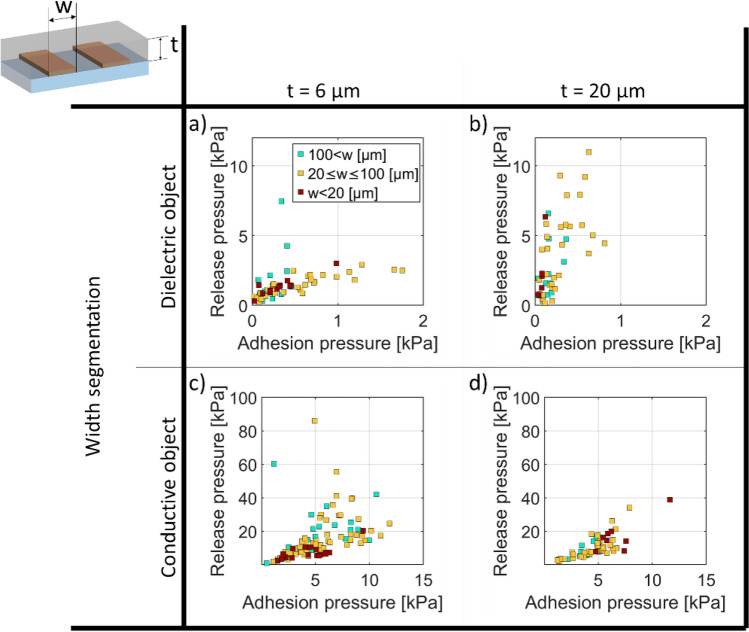
Adhesion versus Release Pressure at 400 V (**a**, **b**) for dielectric objects and (**c**, **d**) conductive object, for an insulator thickness t = 6 μm (**a**, **c**) and 20 μm (**b**, **d**). The data is grouped into three ranges of electrode widths to show trends.

**Figure 8:**
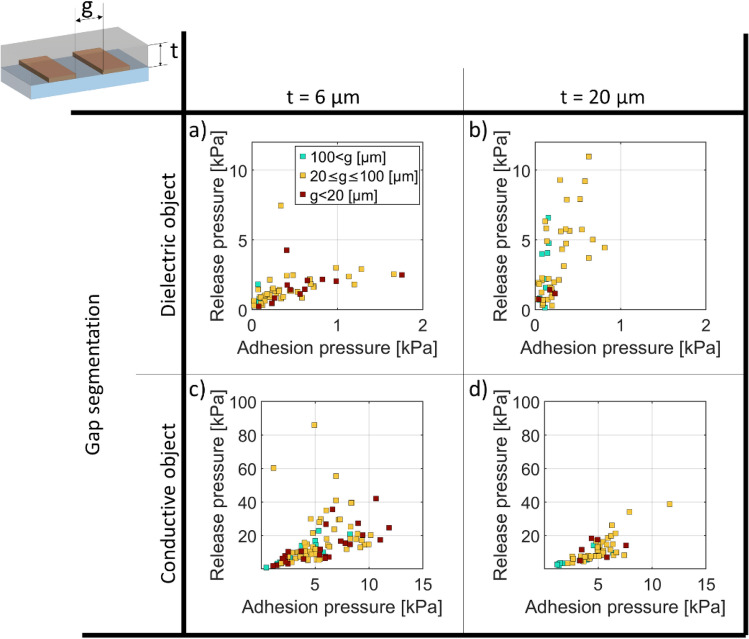
Adhesion versus Release Pressure at 400 V (**a**, **b**) for dielectric objects and (**c**, **d**) conductive object, for an insulator thickness t = 6 μm (**a**, **c**) and 20 μm (**b**, **d**). The data is grouped into three ranges of electrode gaps.

Figures 7 and 8 report release and adhesion pressures for different widths and gaps of the interdigitated electrodes. To show overall trends, we color code widths and gap in 3 bins: smaller than 20 μm, 20 to 100 µm, and greater than 100 μm.

Figure 7a plots adhesion pressure versus release pressure at 400 V, for a dielectric object, with an insulator thickness of 6 µm. The data points are colored according to the three groups of the width of the interdigitated electrodes. The highest adhesion pressures are reached on average for electrode widths between 20 and 100 μm, while the highest release pressures are obtained with width higher than 100 µm. In Fig. 7b) we have the same configuration but for an insulator thickness of 20 µm. The release pressures are similar to the 6 µm case, but the adhesion pressures are smaller.

Figure 7c is similar to 7a but for a conductive object. The highest adhesion pressures are reached on average for widths higher than 20 μm. Figure 7d plots the adhesion pressure versus release pressure at 400 V, for a conductive object, with an insulator thickness of 20 µm. The maximum adhesion and release pressures are reached in average for width lower than 100 μm. Increasing the insulator thickness confined the electric field inside of the insulator and reduces the effect of the width on the EA pressure.

Figure 8a plots the adhesion pressure versus release pressure at 400 V, for a dielectric object, with an insulator thickness of 6 µm. The data is grouped into 3 ranges of interelectrode gaps. Maximum adhesion and release pressures are reached on average for gaps lower than 100 μm. In Fig. 8b) we have the same configuration but for an insulator thickness of 20 µm. Both maximum adhesion and release pressures are reached in average for gap between 20 and 100 μm. Figure 8c is similar to 8.a but for a conductive object. The highest adhesion pressures are reached on average for gaps lower than 20 μm. Finally, Fig. 8d plots the adhesion pressure versus release pressure at 400 V, for a conductive object, with an insulator thickness of 20 µm. The maximum adhesion and release pressures are reached in average for width lower than 100 μm. Increasing the insulator thickness confined the electric field inside of the insulator and reduces the effect of the width on the EA pressure.

It is clearly seen in Fig. 6 that conductive objects have higher EA pressures than dielectric objects, for a given voltage and electrode geometry. Comparing Figs. 7 and 8, for a dielectric object, decreasing the insulator thickness reduces on average the release pressure and increases the adhesion pressure. In the case of a conductive object, decreasing the insulator thickness increases both adhesion and release pressure.

The scatter in our data is not due to charge injection: we know this because we take a force–displacement curve at 0 V before and after every measurement, and can thus verify the absence of residual pressure. The scatter may be in large part due to dust, and hence to an additional spacing between patch and object. To minimize this, we cleaned both the EA pad and the object with lint free wipes before each measurement, but the measurement was done in a standard lab environment (not in a cleanroom).

For a conductive object, adhesion pressures range from 1 to 15 kPa and release pressures range from 1 to 100 kPa. No clear difference is seen between insulator thickness t of 6 µm and 20 µm. For dielectric object, adhesion pressures range from 0.01 to 2 kPa and release pressures from 0.1 and 10 kPa. Higher release/adhesion pressure ratio are seen for an insulating layer of 20 µm compared to 6 µm.

The ratio of release to adhesion pressure is bounded between 1 and 10 for the conductive objects, and between 1.5 and 50 for the dielectric ones, showing the importance of surface forces, charge injections, and possible longer-time scale polarization.

## Conclusion

Our experimental method is unique in measuring both pre-contact EA “remote” forces as well as the larger and more widely reported post-contact release forces. When using EA for manipulation and locomotion, the release force is the practically useful value. The release force however depends on contact time and includes charge injection and surface forces, making it more difficult to tease out the effects of electrode geometry.

Over a wide range of samples, we measured release pressures at 400 V from 0.1 kPa to 100 kPa, similar to the pressures reported in the literature at voltage of several kV, but with larger electrode gaps than ours. The adhesion pressure values cannot be directly compared to prior work on EA because our experiment is the first to measure the electro-adhesive pressure during the adhesion phase, rather than the higher force observed in the detaching phase.

We measured the time dependence of EA pressures, and the resulting residual pressures. The longer one remains in contact with a DC voltage on, the higher the EA release pressure will be, and the higher the residual pressures due to charge injection and remanent polarization will be, lasting 10 to 20 min after a measurement at 1200 V. For applications such as grippers, the drive waveform could be dynamically switched between DC and AC to allow the maximum grasping force in DC while then obtaining minimal residual pressures of AC for quick release.

Complementing standard EA test benches that report only the release pressure, we extended in this paper the measurement methodology to also quantify the pre-contact adhesion force, which excludes contact forces or time dependent interactions with the objects. We used this test bench to study the impact on EA of different object nature (conducting vs. insulating), insulator thickness, and also of different waveforms (e.g., AC or DC). The influence on EA pressure of other parameters such as materials permittivity, roughness, humidity and others could also be studied using this method.

The devices reported in this article were fabricated on rigid substrates, as it was easier to perform photolithography on glass wafers. EA is however often exploited on flexible or even stretchable substrates. We expect all conclusions to hold on curved surfaces when the EA patch is sufficiently compliant to match the object shape.

## Supplementary Information

Supplementary Figures.

## Data Availability

Data for all tested samples and conditions are available in a Zenodo repository. (https://doi.org/10.5281/zenodo.6417174).
