# Supplementary material for: Measuring electro-adhesion pressure before and after contact
Source: Sci Rep. 2023 Jul 20;13:11768. doi: 10.1038/s41598-023-38872-6 (PMC10359345; doi:10.1038/s41598-023-38872-6)
Supplement: Supplementary file 1 — Supplementary Figures. [file 41598_2023_38872_MOESM1_ESM.docx]

**Measuring Electro-Adhesion Pressure before and after contact**

Sylvain Schaller* and Herbert Shea

Sylvain Schaller* (ORCID: 0000-0002-2933-7825),

Prof. Herbert Shea (ORCID: 0000-0003-3527-3036)

Soft Transducers Laboratory , Ecole Polytechnique Fédérale de Lausanne (EPFL)
Rue de la Maladière 71b, CH-2000 Neuchâtel, Switzerland

E-mail: [sylvain.schaller@epfl.ch](mailto:sylvain.schaller@epfl.ch)

**Supplementary Information**

## **S1. EA patch wafer and dimensions**


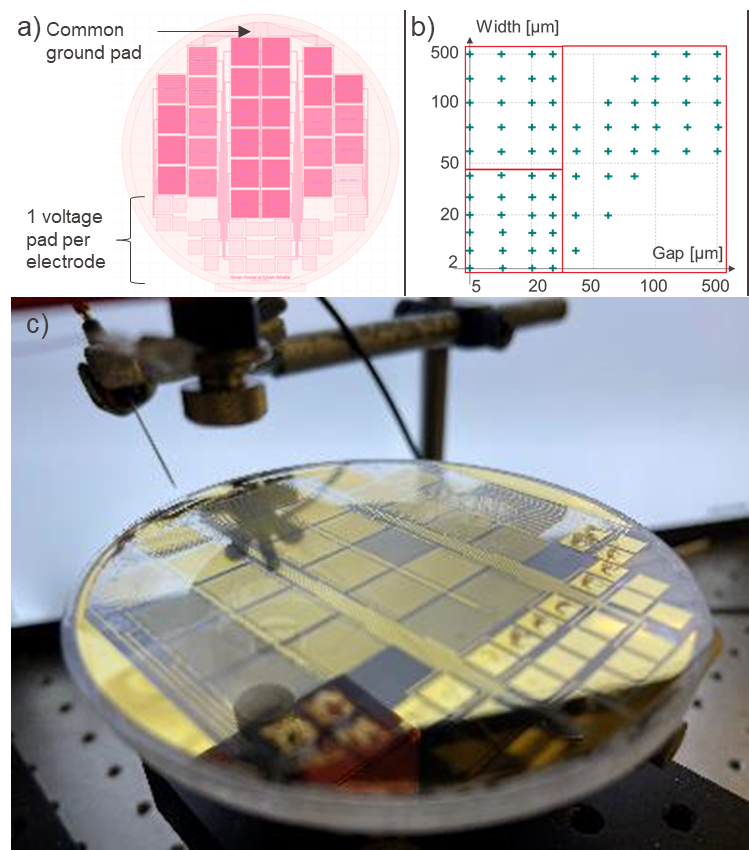


**Supplementary Fig S1.** a) Layout of the electro-adhesive patches on a 4” glass wafer b) Green crosses indicate the width and gaps of the fabricated electrodes, split in 3 wafer layouts (red boxes) c) Photograph of a fabricated wafer on the test bench

We created 3 different wafer layouts, each with 30 EA patches, see Fig S1a and S1b above. Wafer Design A has the smallest electrode dimensions: widths from 2 µm to 30 µm and gaps from 2 to 30 µm. Wafer Design B has small gaps and larger widths (widths from 40 µm to 500 µm and gaps from 2 µm to 30 µm). Wafer Design C has the larger gaps and widths (width from 40 µm to 500 µm and gaps from 40 µm to 500 µm).

We fabricated 3 wafers with designs A, 3 wafers with design and B, and 2 wafers with design C. We then coated each wafer with either 6 µm or 20 µm of P(VDF-TrFE-CTFE). We coated 2 wafers each of design A and B at 6 µm thickness and one at 20 µm thickness. For Design C we coated one wafer at 6 µm and one wafer at 20 µm. This corresponds to 240 fabricated EA patches. All the patches with 2 µm gaps had short circuits or failed once a voltage was applied. 58 other patches failed for various reasons related to fabrication, leaving 182 EA patches used for the experiments.

## **S2. EA patch Fabrication process**

100 mm diameter Borofloat glass wafers were used as substrates.

The first steps are cleaning the wafer in Piranha solution, followed by O_2_ plasma surface treatment. Next is metal evaporation (20 nm of Cr and 80 nm of Au), followed by the deposition of 600 nm of photoresist. The photoresist was exposed by direct laser engraving. After development, Ion Beam Etching is used to etch the Au and Cr layers. The last step is to strip the resist with O_2_ plasma, remover and, if necessary, few more minutes of O_2_ Plasma.

| Step # | Process description | Cross-section after process step |
| --- | --- | --- |
| 01 | Clean with Piranha solution  Treat with O_2_ plasma |   Substrate: Glass Borofloat |
| 02 | Metal Evaporation  Metal : Cr + Au |  |
| 03 | Resist deposition |  |
| 04 | Direct laser writing + Development |  |
| 05 | Ion Beam Etching |  |
| 06 | Strip resist  O_2_ Plasma |  |
| 07 | P(VDF-TrFE-CTFE) deposition |  |

**Supplementary Fig S2.** Wafer-scale microfabrication process for the EA patches

**S3. Flatness of conductive and dielectric objects**

The surface roughness of both conductive and dielectric objects was determined using a laser scanning microscope (Keyence AG, 3D Laser Scanning Confocal Microscope. VK-X1100. 3 Lenses, 10X, 20X and 50X).


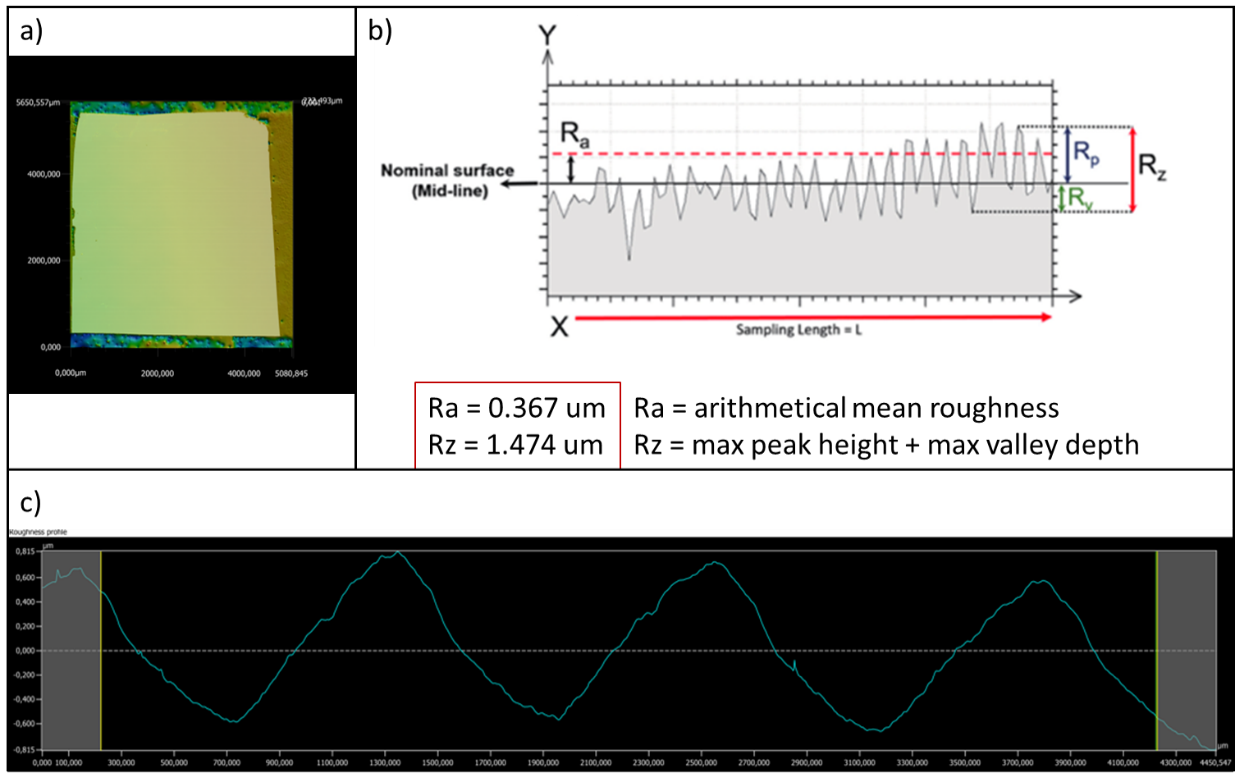


**Supplementary Fig S3.** Scan of surface and roughness of the object using a confocal microscope.

Figure S3a shows an image of the gold-coated conducive object taken using a confocal microscope. Fig S5b shows different surface roughness indicators and their values for our sample. Fig S5c is the cross-section corresponding to the red dashed line in fig S5a.

The conductive object has an R_a_ of 0.367 µm and a R_z_ of 1.474 µm,

## **S4. Object to Patch Angular alignment tolerance determination**

Angular misalignment between the rigid object and the rigid electrodes could significantly change the measured EA pressure, as the misalignment will leads to an air gap. Great care was therefor taken to align the object and the patch before each measurement campaign to ensure a sub-µm air gap.

Fig S4 shows measured release EA pressure vs. angle on one axis at 100V, 200V, 300V and 400V. The EA pressure is reduced by almost 30% for a misalignment of 0.15° on one axis.

Prior to measuring EA pressure as reported in the main manuscript, we carefully set the tilt on both axes by measuring pressure vs. both angles to find the angles that gave the highest pressure.


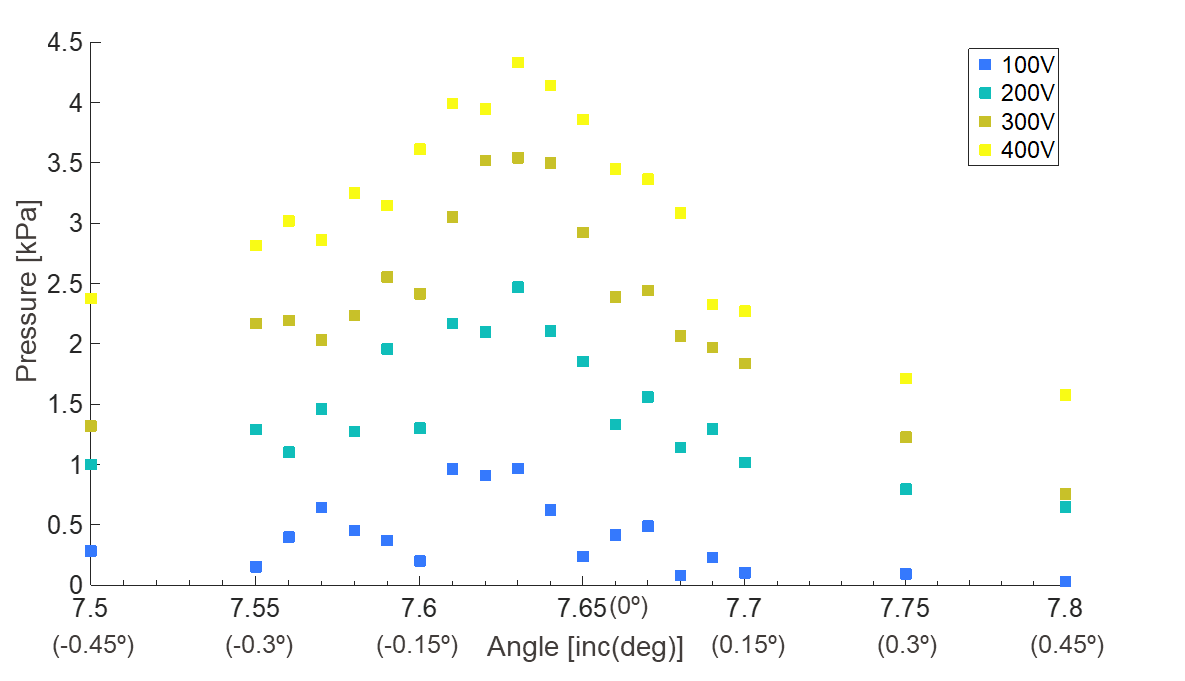


**Supplementary Fig S4.** EA Pressure versus absolute angle of the wafer. The maximum pressure is achieved for best alignment between the object and the patch

## **S5. EA pressure vs AC Frequency**

We characterized EA pressure vs. bipolar AC frequency

For this measurement, we used a patch of electrodes interdigitated width and gap of 100 μm, with 20 μm insulator thickness, a dielectric object and a voltage of 1200 V to prevent a breakdown between the electrodes.

To allow for fast release, one prefers a frequency high enough to minimize charge injection. However, too high a frequency could prevent full polarization of the dipoles. We performed a frequency sweep from 0.1 Hz to 1 kHz and measured the adhesive pressure for a dielectric object.


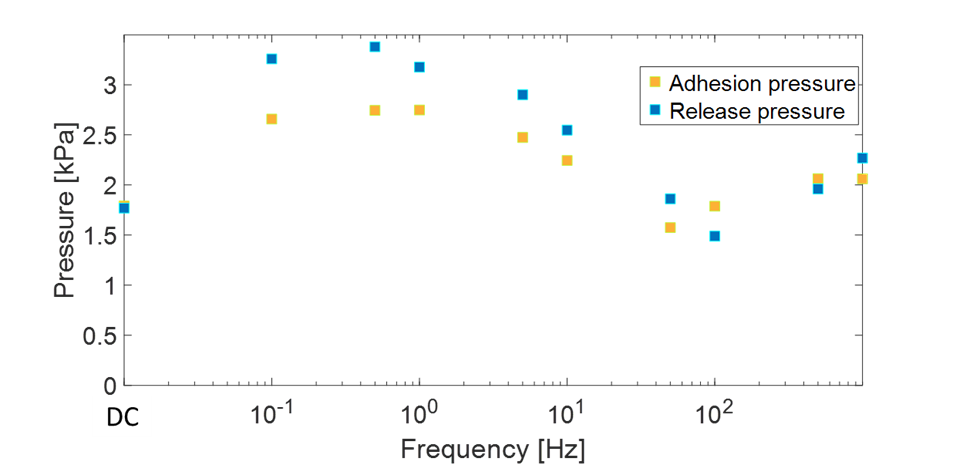


**Supplementary Fig S5** Adhesion and Release pressure vs. frequency for a dielectric object at 1200 V.
